# Progress and gaps in poliovirus immunity: Evidence from a serological survey of children aged 6-23 months in high-risk districts of Pakistan

**DOI:** 10.1038/s41541-025-01352-1

**Published:** 2025-12-30

**Authors:** Imtiaz Hussain, Ahmad Khan, Muhammad Umer, Muhammad Sajid, Haider Abbas, Muhammad Masroor Alam, Muhammad Anwar-ul Haq, Altaf Bosan, Rehan Hafiz, Jeffrey Partridge, Sajid Soofi

**Affiliations:** 1 Centre of Excellence in Women and Child Health, The Aga Khan University, Karachi, Pakistan; 2 Department of Pediatrics & Child Health, The Aga Khan University, Karachi, Pakistan; 3 National Institute of Health, Islamabad, Pakistan; 4 Polio National Emergency Operations Center, Govt of Pakistan, Islamabad, Pakistan; 5 Polio Program, Global Development, Bill & Melinda Gates Foundation, Seattle, USA

**Keywords:** Diseases, Health care, Immunology, Medical research, Microbiology

## Abstract

Wild poliovirus remains endemic in Pakistan and Afghanistan despite global progress. We quantified immunity to poliovirus types 1–3 among children aged 6–23 months in 44 high-risk districts (2022–2023) using a cross-sectional serosurvey with probability proportional to size (PPS) cluster sampling. We enrolled 20,680 children (10,112 aged 6–11 months; 10,568 aged 12–23 months). Seroprevalence among 6–11-month-olds was 94.5% (type 1), 44.6% (type 2), and 88.0% (type 3); among 12–23-month-olds, it was 95.9%, 53.8%, and 91.2%, respectively. Type 1 seropositivity was highest across provinces; type 3 exceeded 90% except in Balochistan and KP; type 2 was lowest everywhere. Younger children have lower immunity. In multivariable models, residence in Balochistan predicted reduced seroprotection (AOR 0.178, 95% CI 0.066–0.484); older age (AOR 1.356, 1.161–1.583), full immunization (AOR 2.004, 1.643–2.444); and receiving <4 oral poliovirus vaccine (OPV) doses showed higher odds (AOR 1.25, 1.021–1.529) of seroprotection. Wealth showed a non-linear association. Gaps in types 2–3 warrant stronger routine immunization, expanded inactivated polio vaccine (IPV), and tailored supplementary immunization activities (SIAs).

## Introduction

Poliomyelitis remains a significant public health concern despite remarkable progress towards its global eradication. Caused by the poliovirus, an enterovirus mainly spread through the fecal-oral route, it can result in permanent paralysis and, in severe cases, death^1^. Following the launch of the Global Polio Eradication Initiative (GPEI) in 1988, there has been a decrease of more than 99% in the global incidence of poliovirus. However, Pakistan and Afghanistan continue to be among the last two countries where the wild poliovirus type 1 (WPV1) is still being transmitted^2,3^. In Pakistan, WPV1 cases have been continuously reported over the years. In 2022, 20 WPV1 cases were reported in the country, followed by 6 cases in 2023, 74 cases in 2024, and 24 cases as of September 2025^4^. Circulating vaccine-derived poliovirus type 2 (cVDPV2) outbreaks have also affected the country in recent years, with 165 cases reported between July 2019 and April 2021^4^. Due to outbreak-response efforts and the nationwide rollout of the monovalent oral poliovirus vaccine type 2 (mOPV2) under the Global Polio Eradication Initiative (GPEI), no cVDPV2 cases have been reported since April 2021. The country’s national polio eradication strategy now combines routine immunization with supplementary immunization activities (SIAs), conducted proactively in high-risk districts and reactively in response to the detection of either wild or vaccine-derived polioviruses. These strategies have been essential in maintaining population immunity and preventing further type 2 outbreaks in Pakistan^5^.

Sustained progress towards polio eradication has fundamentally reshaped population immunity profiles, yet pockets of susceptibility persist where routine immunization and supplementary campaigns have been unevenly distributed^6–8^. Serological surveys provide programmatically actionable estimates of functional protection as they directly measure neutralizing antibodies through standardized poliovirus neutralization assays, revealing gaps that administrative coverage of campaign tallies may miss^9–11^. Evidence from globally diverse settings, including China, Germany, the United States, the Democratic Republic of Congo (DRC), Nigeria, and Cameroon, consistently shows subnational and age-specific variation in seropositivity for poliovirus with implications for micro-planning, catch-up strategies, and the design of outbreak responses^6–8,11–13^.

Type-specific immunity remains central in the bivalent oral poliovirus vaccine (bOPV) plus inactivated poliovirus vaccine (IPV) era, particularly for poliovirus type-2 following the global switch from tOPV to bOPV in 2016. Studies from Vietnam illustrate the role of IPV in closing the type-2 immunity gaps after the switch, aligning with the needs of programs confronting cVDPV2 risk^14^. Urban and special-population studies have further demonstrated how local immunization histories, migration, and occupational exposures shape antibody profiles; for example, healthcare professionals in Brazil and newly resettled refugees in Denmark exhibited distinct seroprotection patterns that informed targeted interventions^9,10^. In West and Central Africa, serosurveys conducted among adults and children have provided insights into residual susceptibility in complex delivery environments, thereby informing resource prioritization and guiding the refinement of supplementary immunization activities^6,12,13^.

Methodological considerations are also crucial factors in interpreting seroprevalence. A comparative evaluation of virus-neutralization assays highlights variability across platforms. It underscores the value of harmonization for non-polio enteroviruses, a lesson that translates to poliovirus serology, that host and delivery-system factors, such as seasons of vaccination, prior exposures, and cohort effects, can influence measured antibody levels, reinforcing the need for locally generated estimates rather than reliance on global averages^15–18^. Although outside the scope of poliovirus, studies of other vaccine-preventable and viral infections (e.g., varicella disease burden, pertussis, mumps, hepatitis B) and even enterovirus surveillance in animal reservoirs collectively demonstrate how serology guides prevention policy when routine data are insufficient^18–21^.

In Pakistan, districts categorized as very high-risk districts (VHRDs) concerning polio cases share characteristics commonly observed in other high-risk settings, including population mobility, missed opportunities for routine vaccination, hard-to-reach communities, and a recurrent need for targeted campaigns. In this context, a seroprevalence survey among children in VHRDs was essential to quantify seroprotection against poliovirus types 1, 2, and 3 after years of bOPV/IPV use and targeted responses, as well as risk factors associated with immunity levels. It was also imperative to generate district-level evidence to direct outreach and microplanning. By situating Pakistan’s estimates within the context of international experiences, including urban serosurveys, refugee populations, and post-switch evaluations, this study aims to provide decision-oriented immunity maps that support risk-based strategies for the eradication of poliovirus^7,8,14,17,22^.

## Results

A total of 20,680 children were included (10,112 aged 6–11 months and 10,568 aged 12–23 months); the mean age was 13.5 ± 5.3 months (8.9 ± 1.7 and 17.9 ± 3.5 months for the two cohorts, respectively). Gender distribution was balanced (52.0% male overall; 51.5% and 52.4% in the younger and older cohorts, respectively). Most participants were from Khyber Pakhtunkhwa (KP) (37.0%), followed by Sindh (27.2%), Balochistan (22.4%), Punjab (11.2%), and ICT (2.3%). The provincial distribution was similar across age groups. Over half lived in households with ≥7 members (53.4%). Socioeconomic status was evenly distributed across wealth quintiles ( ~ 20% in each). Overall, maternal literacy was recorded at 63.7% (Table 1).

**Table 1:** Demographic indicators, residence, and vaccination history of the study population

	Total	6–11 months	12–23 months
	N = 20680	N = 10112	N = 10568
**Mean age (months)**	13.5 ± 5.3	8.9 ± 1.7	17.9 ± 3.5
**Gender of Child**			
Male	10,751 (52.0%)	5210 (51.5%)	5541 (52.4%)
Female	9929 (48.0%)	4902 (48.5%)	5027 (47.6%)
**Province**			
Punjab	2307 (11.2%)	1106 (10.9%)	1201 (11.4%)
Sindh	5617 (27.2%)	2739 (27.1%)	2878 (27.2%)
Balochistan	4632 (22.4%)	2342 (23.2%)	2290 (21.7%)
KPK	7654 (37.0%)	3696 (36.6%)	3958 (37.5%)
ICT	470 (2.3%)	229 (2.3%)	241 (2.3%)
**Family size**			
<7	9632 (46.6%)	4802 (49.9%)	4830 (50.1%)
>=7	11048 (53.4%)	5310 (48.1%)	5738 (51.9%)
**Wealth Quintiles**			
Poorest	4116 (19.9%)	2058 (50.0%)	2058 (50.0%)
Poor	4143 (20.0%)	2058 (49.7%)	2085 (50.3%)
Middle	4167 (20.1%)	2028 (48.7%)	2139 (51.3%)
Rich	4132 (20.0%)	1975 (47.8%)	2157 (52.2%)
Richest	4122 (19.9%)	1993 (48.4%)	2129 (51.6%)
**Mother education**			
Literate	7513 (36.3%)	3644 (36.0%)	3869 (36.6%)
Illiterate	13,167 (63.7%)	6468 (64.0%)	6699 (63.4%)
**Child ever received IPV**			
Yes	11409 (55.2%)	4936 (48.8%)	6473 (61.3%)
No	9271 (44.8%)	5176 (51.2%)	4095 (38.7%)
**Total OPV doses (RI** + **SIAs)**			
<4	14201 (68.7%)	7186 (71.1%)	7015 (66.4%)
>=4	6479 (31.3%)	2926 (28.9%)	3553 (33.6%)
**Immunization status**			
Not immunized	3733 (18.1%)	1946 (19.2%)	1787 (16.9%)
Partially immunized	9260 (44.8%)	3860 (38.2%)	5400 (51.1%)
Fully immunized	7687 (37.2%)	4306 (42.6%)	3381 (32.0%)

*KP* Khyber Pakhtunkhwa, *ICT* Islamabad Capital Territory, *IPV* Inactivated Poliovirus Vaccine, *OPV* Oral Poliovirus Vaccine, *RI* Routine Immunization, *SIAs* Supplementary Immunization Activities.

Of the children included in the survey, 55.2% had received IPV, with a higher proportion of children in the 12-23-month age group than in the 6–11-month age group (61.3% vs 48.8%, respectively). Altogether, only 31.3% of children received four or more doses of OPV (33.6% of those 12–23 months old and 28.9% in the 6–11 months age group).

Overall, 37.2% of children had received all age-appropriate vaccines as per the EPI schedule of Pakistan, and 44.8% were partially immunized. A higher proportion of children in the 6–11 months cohort (42.6%) had received all age-appropriate vaccines compared to the 12–23 months cohort (32.0%). A higher proportion of children in the older age cohort were partially immunized (51.1%) compared to those in the younger age cohort (38.2%). District-wise demographic indicators are present in Table S1.

### Seroprevalence of poliovirus types 1, 2, and 3 by age cohort and province

**Fig. 1:**
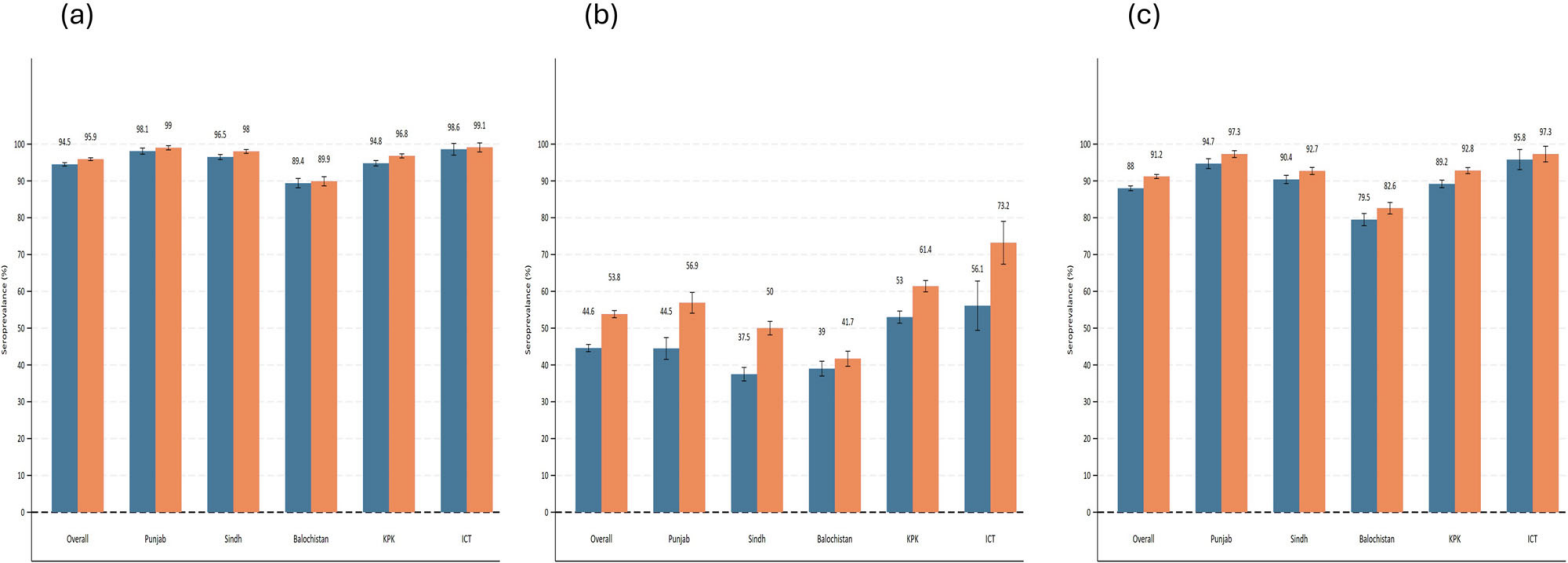
Seroprevalence of poliovirus types 1, 2, and 3 among children aged 6–11 months and 12–23 months across provinces and the Islamabad Capital Territory (ICT). **a** Poliovirus type 1; **b** Poliovirus type 2; **c** Poliovirus type 3. Dark blue bars: Children aged 6–11 months Orange bars: Children aged 12–23 months.

Overall, seroprevalence for type 1 was 94.5% (CI: 94–95) and 95.9% (CI: 95.5–96.3) among children 6-11 months and 12-23 months, respectively. The seroprevalence was uniformly high ( > 94%) across all provinces with minimal age-related differences. On the contrary, type 2 displayed a substantial immunity gap, particularly in younger children, 44.6% (CI: 43.6–45.6), compared to the older age cohort, 53.8% (CI: 52.8–54.8), with the lowest levels in Baluchistan [39% (CI: 37–41) among the younger age cohort, 41.7% (CI: 39.7–43.7) among the older age cohort] and Sindh [37.5% (CI: 35.7–39.3) younger age cohort, and 50% (CI: 48.2–51.8) among older age cohort)] (Fig. 1). Districts in Baluchistan and Sindh had low type 2 immunity compared to districts in other provinces (Table S2).

The prevalence of type 3 seroprotection showed intermediate protection, above 90% for all provinces and both age cohorts except in Balochistan [79.5% (95% CI: 77.8–81.2) for younger age cohort] and [82.6% (CI:81.1–84.2) for older age cohort] and in KP [for younger age cohort 89.2% (CI: 88.2–90.2 compared to 92.8% (CI: 92.0–93.6)] (Fig. 1). Half of the districts in Baluchistan had low type immunity among younger children (Table S2). Overall, immunity was higher for type 1, lower for type 2, and moderate for type 3, with Baluchistan consistently lagging and ICT maintaining the highest immunity across all serotypes.

### Reverse cumulative distributions of neutralizing antibody titers for poliovirus types 1, 2, 3 by age cohort and province

**Fig. 2:**
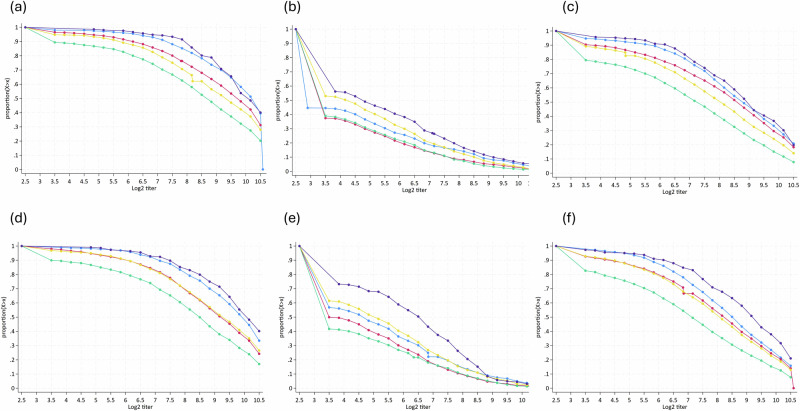
Reverse cumulative distributions of neutralizing antibody titers for poliovirus types 1–3 in children aged 6–11 and 12–23 months by province and Islamabad Capital Territory (ICT). **a** Antibody titers for poliovirus type 1 among children aged 6–11 months; **b** Antibody titers for poliovirus type 2 among children aged 6–11 months; **c** Antibody titers for poliovirus type 3 among children aged 6-11 months; **d** Antibody titers for poliovirus type 1 among children aged 12–23 months; **e** Antibody titers for poliovirus type 2 among children aged 12–23 months; **f** Antibody titers for poliovirus type 3 among children aged 12–23 months. Bright blue line: Punjab; Marron line: Sindh; Forest green line: Balochistan; Yellow line: Khyber Pakhtunkhwa (KP); Dark blue line: Islamabad Capital Territory (ICT).

The strength of immunity was assessed through the reverse cumulative distribution of neutralizing antibody titers for poliovirus types 1, 2, and 3 in children aged 6–11 months (Fig. 2a–c) and 12-23 months (Fig. 2d–f) across the provinces. The farther the right-shifted curves are, the stronger the immunity. Poliovirus type 1 (PV1) exhibits near-universal, high-titer protection across provinces, with ICT and Punjab showing the highest titters and Baluchistan the lowest; older children have slightly higher titters. Poliovirus type 2 (PV2) shows the weakest immunity, with a steep drop in titers after reaching low levels, highlighting significant gaps in Baluchistan and Sindh. In contrast, ICT and KP show comparatively better results, with older children again displaying modestly higher titters. Poliovirus type 3 (PV3) lies between PV1 and PV2, offering generally strong protection but lower than PV1, and Baluchistan consistently lags. Overall, the figure demonstrates robust PV1 and PV3 immunity, but persistent gaps in PV2 immunity, especially among younger children and in Sindh and Balochistan.

### Risk factors associated with poliovirus seronegativity: Univariate and multivariate analysis

We assessed risk factors associated with poliovirus seroprevalence using the univariate and multivariate models. Variables with a *p*-value < 0.25 in the univariate analysis were considered for inclusion in the multivariate model. However, only those with *p*-values < 0.05 were retained in the final model. Sero-negativity was highest in Baluchistan (8.0%) and KP (3.0%) compared to Punjab (1.1%), Sindh (1.7%), and ICT (0.9%). In the adjusted model, children from Baluchistan have significantly lower odds of seropositivity (AOR 0.178, CI: 0.066–0.484; *p* < 0.001). A nonlinear association was found between household wealth and level of seroprotection, with nonsignificant differences between the poorest and richest groups (AOR 1.105, 95% CI: 0.816–1.496, *p* = 0.520). Children in the older age cohort were more likely to be seroprotected compared to the younger age cohort (AOR 1.356, 95% CI: 1.161–1.583, *p* < 0.001). Also, receiving <4 doses of OPV had 1.2 times higher odds of seroprotection (AOR 1.25, 95% CI: 1.021–1.529, *p* < 0.030) compared to receiving >= 4 doses (Table 2).

**Table 2:** Univariate and Multivariate Analysis for Risk Factors Associated with Seroprevalence

			Unadjusted Odds Ratio for Seropositivity	P-values	Adjusted Odds Ratio for Seropositivity	*P*-values
	Any Type Poliovirus					
	Seronegative	Seropositive				
	N = 708	N = 19,409				
**Province**						
Punjab	24 (1.1%)	2231 (98.9%)	0.861 (0.297,2.493)	0.782	0.695 (0.238,2.026)	0.505
Sindh	94 (1.7%)	5414 (98.3%)	0.533 (0.195,1.458)	0.220	0.668 (0.243,1.835)	0.433
Balochistan	362 (8.0%)	4138 (92.0%)	0.106 (0.039,0.285)	<0.001	0.178 (0.066,0.484)	**0.001**
KPK	224 (3.0%)	7194 (97.0%)	0.297 (0.110,0.803)	0.017	0.415 (0.153,1.127)	0.084
ICT	4 (0.9%)	432 (99.1%)	Ref.		Ref.	
**Family size**						
<7	253 (2.7%)	9120 (97.3%)	Ref.			
>=7	455 (4.2%)	10289 (95.8%)	0.627 (0.537,0.733)	<0.001		
**Wealth Quintiles**						
Poorest	146 (3.6%)	3884 (96.4%)	Ref.		Ref.	
Poor	169 (4.2%)	3888 (95.8%)	0.865 (0.690,1.084)	0.207	0.885 (0.702,1.116)	0.302
Middle	186 (4.6%)	3850 (95.4%)	0.778 (0.624,0.971)	0.026	0.679 (0.54,0.854)	**0.001**
Rich	136 (3.4%)	3876 (96.6%)	1.071 (0.845,1.359)	0.570	0.74 (0.579,0.946)	**0.016**
Richest	71 (1.8%)	3911 (98.2%)	2.071 (1.554,2.759)	<0.001	1.105 (0.816,1.496)	0.520
**Mother education**						
Literate	158 (2.2%)	7148 (97.8%)	2.029 (1.696,2.428)	<0.001		
Illiterate	550 (4.3%)	12,261 (95.7%)	Ref.			
**Sex of Child**						
Male	357 (3.4%)	10,079 (96.6%)	1.062 (0.914,1.234)	0.431		
Female	351 (3.6%)	9330 (96.4%)	Ref.			
**Child age**						
6-11 months	409 (4.2%)	9377 (95.8%)	Ref.		Ref.	
12-23 months	299 (2.9%)	10,032 (97.1%)	1.463 (1.257,1.703)	<0.001	1.356 (1.161,1.583)	**<0.001**
**Child ever received IPV**						
Yes	230 (2.1%)	10,892 (97.9%)	Ref.			
No	478 (5.3%)	8517 (94.7%)	0.376 (0.321,0.441)	<0.001		
**Total OPV doses (RI** + **SIAs)**						
<4	505 (3.7%)	13,328 (96.3%)	0.881 (0.747,1.040)	<0.001	1.25 (1.021,1.529)	**0.030**
>=4	203 (3.2%)	6081 (96.8%)	Ref.		Ref.	
**Immunization status**						
Not immunized	280 (7.8%)	3312 (92.2%)	Ref.		Ref.	
Partially immunized	146 (1.6%)	8884 (98.4%)	5.144 (4.195,6.309)	<0.001	3.846 (2.99,4.947)	**<0.001**
Fully immunized	282 (3.8%)	7213 (96.2%)	2.162 (1.824,2.564)	<0.001	2.004 (1.643,2.444)	**<0.001**

Bold values indicate statistically significant results (*p* < 0.05).

*KP* Khyber Pakhtunkhwa, *ICT* Islamabad Capital Territory, *IPV* Inactivated Poliovirus Vaccine, *OPV* Oral Poliovirus Vaccine, *RI* Routine Immunization, *SIAs* Supplementary Immunization Activities.

A clear association was observed between immunization and immunity level: non-immunized children had the highest sero-negativity (7.8%), partially immunized children showed relatively higher protection (AOR 3.846, CI: 2.99–4.947; *p* < 0.001), while being fully immunized (i.e., receiving all age-appropriate vaccines) was significantly associated with higher seroprotection. A fully immunized child is 2 times more protected compared to a non-immunized child (AOR 2.004, 95% CI: 1.643–2.444, *p* < 0.001).

Comparing multivariate models between serotypes revealed a number of predictors that were consistently associated with lower seropositivity for PV1, PV2, and PV3, including residence in Balochistan, larger household size, lack of IPV, and incomplete immunization. However, PV2 followed a different pattern compared to the other serotypes. Fully immunized children (i.e., receiving all age-appropriate vaccines) did not demonstrate higher PV2 seropositivity, and geographic disparities extended beyond Balochistan to include Punjab and Sindh. By contrast, receipt of IPV emerged as a particularly strong and independent predictor for PV2, highlighting the specific determinants of type-2 immunity in the post-switch setting.

## Discussion

This seroprevalence survey, conducted in 44 very high-risk districts of Pakistan, provides one of the most granular assessments of poliovirus immunity among children in recent years. Our results showed that overall population-level immunity remains high for the type I strain, with more than 94% of children showing serological evidence of protection. However, significant variability in immunity levels persists across age groups and regions, and immunity gaps exist for virus types 2 and 3, similar to patterns observed in other endemic regions. The findings highlight both the progress made and the fragility of eradication efforts, especially regarding poliovirus types 2 and 3 among the target age groups.

Our results for type 1 are consistent with findings from other studies in diverse settings. An Afghan serological survey conducted in 2017 also recorded similarly high type 1 immunity in children under two years of age^23^. Additionally, in India, seroprotection for type 1 polio was over 95% in Uttar Pradesh—a polio high-risk region—before the disruption of wild poliovirus transmission^24^. Likewise, in West Africa, serosurveys in Nigeria and Chad also observed greater protection against type 1 in zones where intensive SIAs were conducted^25,26^. These convergent findings suggest that, despite operational weaknesses, frequent OPV exposure has been sufficient to maintain high immunity against type 1 in various settings.

Conversely, our findings of low immunity level for the type 2 strain reflect the global consequence of the tOPV withdrawal in 2016. In the post-switch period, type 2 seroprevalence in Pakistan declined below 50% among children under two years, especially in children with low IPV coverage^3^. A multi-country analysis of poliovirus immunity levels reported that type 2 immunity declined steeply two to three years after the global post-switch, particularly in regions with suboptimal IPV coverage^27^. A similar trend was reported in Nigeria, where type 2 population immunity decreased after the transition, leading to recurrent outbreaks of circulating vaccine-derived poliovirus type 2 (cVDPV2)^28^. A study from Syria showed that large cVDPV2 outbreaks after tOPV withdrawal unveiled immunity gaps among children under two years of age, via low IPV uptake and immunization disruption due to conflict^29^.

Our reverse cumulative titer curves for type 2 immunity showed that protection in the target regions is weak in strength and low in coverage, with much lower median titers. These lower type 2 titers likely reflect suboptimal IPV coverage and waning systemic immunity in the post-switch period, factors that influence population susceptibility and transmission risk, but do not directly reduce neutralizing antibody levels. Findings from a randomized controlled trial in India indicated that a single dose of IPV is sufficient to enhance type 2 immunity. However, restoring population immunity requires higher coverage and, in specific settings, targeted use of monovalent OPV^30^. The low median titers in our study, particularly in more classically high-risk settings, suggest that systemic immunity is suboptimal in these groups, presumably due to an incompletely vaccinated population, missed doses, or waning antibody levels. Combined, these double vulnerabilities underscore the heightened risk for children in such settings to be exposed to poliovirus circulation. Regarding the durability of protection, IPV has been shown to induce long-term systemic neutralizing antibodies, whereas OPV provides both mucosal and systemic immunity with somewhat more rapid waning of humoral antibody levels in the absence of repeated exposure or booster doses from supplementary campaigns^31–33^. This difference in durability reflects the need for continued high OPV coverage alongside timely administration of IPV to maintain population immunity, particularly in areas at risk of transmission.

Seroprotection against poliovirus type 3 in our study was lower compared to the type 1 immunity level, and showed extensive provincial variation, with the lowest rates in Baluchistan. These findings are consistent with global evidence that type 3 immunity lags type 1, even in countries with higher routine immunization coverage. The type 3 component of OPV is less immunogenic than the type 1 component, and seroconversion requires multiple doses. In India and Pakistan, type 3 seroprevalence generally lagged behind type 1 by 5–10 percentage points, both due to its diminished immunogenicity and irregular SIA quality^24^. These disparities have also been observed in Nigeria and South Africa, where type 3 immunity consistently fell below the level required to break transmission^25,34^. Comparisons highlight that type 3 is an ongoing issue across the world, where not only routine vaccination but also high-quality SIAs are required in low-coverage pockets of the population.

The gradient of age in our findings, with older children aged 12–23 months having higher immunity to all serotypes, reflects cumulative exposure from routine immunization and campaigns. Such age effects have been observed earlier in India, Nigeria, and Afghanistan, where younger children typically exhibit lower seroprevalence until more doses of OPV are administered^25,35,36^. Similarly, cross-sectional studies in China and Cameroon have also reported the same trends^11,13^. Also, Pakistan-specific studies have consistently demonstrated similar trends^37,38^. The continuity of these trends at the regional levels is a sign of missed opportunities during the first year of life, reflecting gaps in routine immunization, integration of services with maternal and child health, and the effectiveness of SIAs. They emphasize the need for effective and timely routine immunization to fill immunity gaps among the most vulnerable age groups.

Our analysis identifies key demographic and programmatic factors influencing poliovirus seroprevalence in Pakistan. Geographic inequity was evident in Balochistan, whereby children had significantly lower odds of seropositivity compared with other provinces, even after adjusting for confounding factors. This finding aligns with sustained concerns about programmatic challenges in Balochistan, including security-related restrictions, weak health infrastructure, and widespread population mobility, which collectively undermine routine immunization delivery and quality of SIAs^39^. These factors likely led to the consistently lower seropositivity observed in Balochistan, especially for type 2, where limited routine immunization coverage, lower IPV uptake, hard-to-reach populations, and variable quality SIAs limit opportunities for children to develop sufficient immunity. These geographic variations in immunity have also been reported in Nigeria and Afghanistan, where ongoing reservoirs of incompletely immunized children have caused poliovirus transmission despite very high immunization coverage at the national level^35,36^. Our comparison of subtype-specific multivariate models showed that several predictors (residing in Balochistan, larger household size, absence of IPV, and incomplete immunization) were consistently associated with lower seropositivity across all three serotypes. However, the pattern for PV2 differed considerably from PV1 and PV3. For PV2, fully immunized children (i.e., children who had received all age-appropriate vaccines) were no more likely to demonstrate higher seropositivity, and geographic disparities extended beyond Balochistan to include Punjab and Sindh. IPV emerged as a particularly strong and independent predictor of PV2 immunity, reflecting the post–tOPV withdrawal context in which IPV is the sole antigen for type 2 protection. Similar findings have been reported from Nigeria, where seropositivity for type 2 was significantly higher among children who had received an IPV dose compared with those who had not^35^. These subtype-specific differences reflect unique epidemiologic and programmatic challenges in maintaining type 2 immunity.

Maternal literacy was highly correlated with seroprotection in our unadjusted model, and mothers whose children were literate were likely to be tested seropositive. This finding aligns with earlier studies conducted in Pakistan and other low- and middle-income countries, which have demonstrated that caregiver education improves health-seeking behavior and vaccine coverage^36,40,41^. While attenuated in the adjusted model, the direction of the association serves to emphasize the continued influence of social determinants on immunity outcomes.

Household wealth had a nonlinear effect on seroprotection level in our study. Children in the wealthiest quintile had no appreciably greater odds of seropositivity compared to those in the poorest quintile. However, those in the more affluent and intermediate quintiles had decreased odds. The contradictory trends are hypothesized to indicate that the economic slopes of immunity to polio might be more complex than those for other childhood vaccines, perhaps because of the widespread use of mass campaigns that span socioeconomic lines. However, data from international eradication contexts indicate that socioeconomic disadvantage remains a persistent risk factor for under-immunization, particularly in areas where routine service availability is limited^25,36,41^.

Vaccination history was one of the major predictors of seroprotection. While this association between vaccination and higher seropositivity is expected, our findings underscore that regular participation in immunization services has a critical role in maintaining population immunity. Children who were reported to have received all age-appropriate vaccines had significantly higher odds of seropositivity, suggesting a cumulative effect of complete immunization. Receipt of all-age-appropriate vaccines, including vaccines such as the Pentavalent series, would likely reflect households with better access to, and better acceptance of, immunization services. This may further increase their chances of getting OPV both from routine and supplementary immunization activities. Thus, completion of routine immunization schedules might act as a proxy indicator for poliovirus immunity, and strengthening overall EPI performance is imperative to sustain eradication gains. Similar findings were reported in a study from Nigeria, where vaccination status was associated with seropositivity for poliovirus^25^.

Our findings indicate that a higher percentage of children in the younger age cohort had received all age-appropriate vaccinations compared to the older age cohort, probably reflecting recent improvements in immunization coverage and the cumulative effect of ongoing supplementary immunization activities. In contrast, some of the older children may have missed doses due to campaign interruptions during the COVID-19 pandemic or population mobility, leading to lower completion rates in the 12–23-month cohort. Contrary to previous studies, our analysis revealed that children who reported receiving fewer than four doses of OPV had marginally higher adjusted odds of seropositivity compared to those who received four or more doses^37,42^. This seeming paradox may have resulted from parental recall bias and misclassification of vaccine doses during recall. It may also indicate differences in the quality or intensity of supplementary campaigns, where fewer but more intense exposures produced comparable or stronger immune responses. This inconsistency needs closer attention, most importantly by validation of vaccination history against independent records.

Together, these results show that a dynamic interplay of geographic, demographic, and programmatic factors shapes Pakistan’s poliovirus immunity. Structural disparities, the impact of maternal education, and the priority of universal immunization all make it clear that eradication is both a social and biomedical challenge. Continued progress will entail not just high rates of coverage through routine and supplemental campaigns but also interventions aimed at eliminating regional and social disparities that continue to propel transmission.

This study is not without limitations. Its cross-sectional nature constrains causal inferences from individual-level changes in immunity. The history of vaccination was partially ascertained by caregiver report in the absence of vaccination cards. This is likely to result in the unintentional recall bias for vaccination status. Neutralizing antibody titers are a measurement of humoral immunity. Still, they will not necessarily capture mucosal immunity that is essential for breaking the transmission of poliovirus, especially when using the oral polio vaccine. In addition, the neutralization assay measures total poliovirus-neutralizing antibodies and cannot distinguish between antibodies derived from vaccination and those resulting from natural infection. Contextual factors, such as seasonal migration, access issues, and overlapping supplementary immunization activities during the survey period, could have affected both vaccination exposure and seroprevalence measured, possibly limiting generalizability. Despite this, our findings reveal prevalence and inequalities in poliovirus immunity in high-risk regions of Pakistan, providing evidence for targeted programmatic interventions.

Our findings underscore the necessity of addressing immunity gaps in types 2 and 3 in Pakistan’s polio program, especially for younger children in the high-risk areas. Enhancing the quality and reach of OPV campaigns, expanding IPV coverage, and integrating immunization with maternal and child health services are crucial for ensuring protection in the early years of life. Simultaneously addressing vaccine hesitancy and building trust through sustained community involvement would help to strengthen the approach towards poliovirus eradication in the country.

## Methods

### Study design and participants

We conducted a district-specific cross-sectional seroprevalence survey across 44 very high-risk districts of Pakistan, identified by the National Emergency Operation Center (NEOC) due to persistent poliovirus detection in 2022 (Figure S1 & Table S1). These districts served as geographical strata for this survey. Children were stratified into two age cohorts (6–11 months and 12–23 months) and included in the survey.

### Sample Size

The sample size for this survey was calculated based on an assumed true seroprevalence of 90% and a 95% confidence interval with a ± 5% margin of error. A design effect of 1.5 and an anticipated response rate of 90% were considered. This resulted in a required sample size of 210 children per age cohort per stratum (district), totaling 420 children from each district across both age cohorts.

### Sampling Procedure

To draw the sample from the target districts, a 1 × 1 km grid was generated over each district using ArcGIS (version 10.2.2). Grids with no residential population were excluded. Only grids with a population greater than 100 and neighboring grids with more than 300 individuals were combined to form the sampling frame. For proportional representation, stratified random sampling was employed at the tehsil (sub-districts) level. In each tehsil, probability proportional to size (PPS) was applied so that grids with larger populations had a higher chance of being selected. A total of 25 clusters were randomly selected per district, with an additional five clusters per district identified as backups. Overall, 1100 clusters were included in the survey design from the 44 target districts, with 125 backup clusters.

### Data Collection

Web-based district maps were generated using Google’s application programming interfaces (APIs) and uploaded onto handheld devices to guide data collectors. Each map displayed cluster boundaries, cluster extension boundaries, and centroids to ensure accurate navigation to households. Data collection was completed in two phases across the provinces between September 2022 and October 2023. A structured questionnaire was designed to be administered to mothers or caregivers of the target children, collecting information on household demographics and socioeconomic status, as well as child history, including vaccination status, for all children enrolled in the survey. Data on vaccination were recorded from vaccination cards, and in the absence of cards, data were collected through mother/caregiver recall. Along with the data, 2 mL of venous blood was obtained from each child by a trained phlebotomist. During the surveys, households that were locked or those that refused to participate were not replaced with other households.

The district data collection team consisted of two female data collectors, two female phlebotomists, and a male team leader. The team received a comprehensive training, conducted over five days, led by experienced investigators and faculty members from Aga Khan University (AKU).

In this study, “fully immunized” refers to children who had received all age-appropriate vaccines according to Pakistan’s Expanded Programme on Immunization (EPI) schedule at the time of the survey. These vaccines include Bacillus Calmette-Guérin vaccine (BCG), OPV0, OPV1–3, Pentavalent1–3, Pneumococcal Conjugate vaccine (PCV1–3), IPV, and Measles-containing vaccine (MCV1). The definition excluded vaccines that are recently rolled out but not uniformly being administered across all provinces, such as hepatitis B (Hep-B) at birth, ROTA 1 at six weeks, ROTA 2 at 10 weeks, and Typhoid conjugate vaccine (TCV) at nine months.

### Laboratory methodology

After blood collection from a child, each sample was centrifuged, and the serum was isolated and put into sterile, labeled cryovials. The cryovials were stored in a cold box with ice packs and sent immediately to the nearest laboratory collection point of AKU. From these collection points, the samples were transported to the Nutrition Research Laboratory at AKU in Karachi. During the transportation, the temperature of the samples was monitored using a digital thermometer attached to the lid of the ice box. At the laboratory at AKU, two distinct aliquots were prepared; one was kept as a backup, and the other was sent to the National Institute of Health (NIH), Pakistan, for neutralization assay examination^43^. For this study, seropositivity was defined as a titer of poliovirus neutralizing antibody greater than 1:8^31^.

### Statistical analysis

Seroprevalence was estimated for poliovirus types 1, 2, and 3 by age cohort, child sex, and province. Point estimates were reported at 95% confidence intervals to account for the survey design effect. Immunity gaps were explored by stratifying results by demographic and geographic characteristics.

Bivariate analyses were performed to examine the associations between seronegativity and selected covariates, including child age, sex, province of residence, maternal literacy, socioeconomic indicators, and vaccination history, such as receipt of IPV and full immunization status. For categorical variables, the Chi-square test was used with a significance level set at p < 0.05.

Multivariate logistic regression models were fitted to identify independent predictors of seronegativity for each poliovirus type. Adjusted odds ratios (AORs) at 95% CIs were reported. Model specification considered potential confounders identified a priori from the literature, as well as variables significant at *p* < 0.25 in univariate analysis.

Sensitivity analyses were performed to assess the robustness of estimates by excluding children with incomplete vaccination histories and stratifying the results by urban–rural residence. The analysis was conducted using STATA version 18^44^.

### Ethical approval

We obtained ethical approval from the Ethical Review Committee of Aga Khan University, Pakistan (2022-7646), and the National Bioethics Committee, Pakistan (NBC-814). Informed consent was obtained from the caregivers of the participants included in the study.

## Supplementary information

Supplementary Information

## Data Availability

The data will be made available upon request.
