# Supplementary material for: Progress and gaps in poliovirus immunity: Evidence from a serological survey of children aged 6-23 months in high-risk districts of Pakistan
Source: NPJ Vaccines. 2025 Dec 30;11:31. doi: 10.1038/s41541-025-01352-1 (PMC12852092; doi:10.1038/s41541-025-01352-1)
Supplement: Supplementary file 1 — Supplementary Information [file 41541_2025_1352_MOESM1_ESM.docx]

**Supplementary materials**


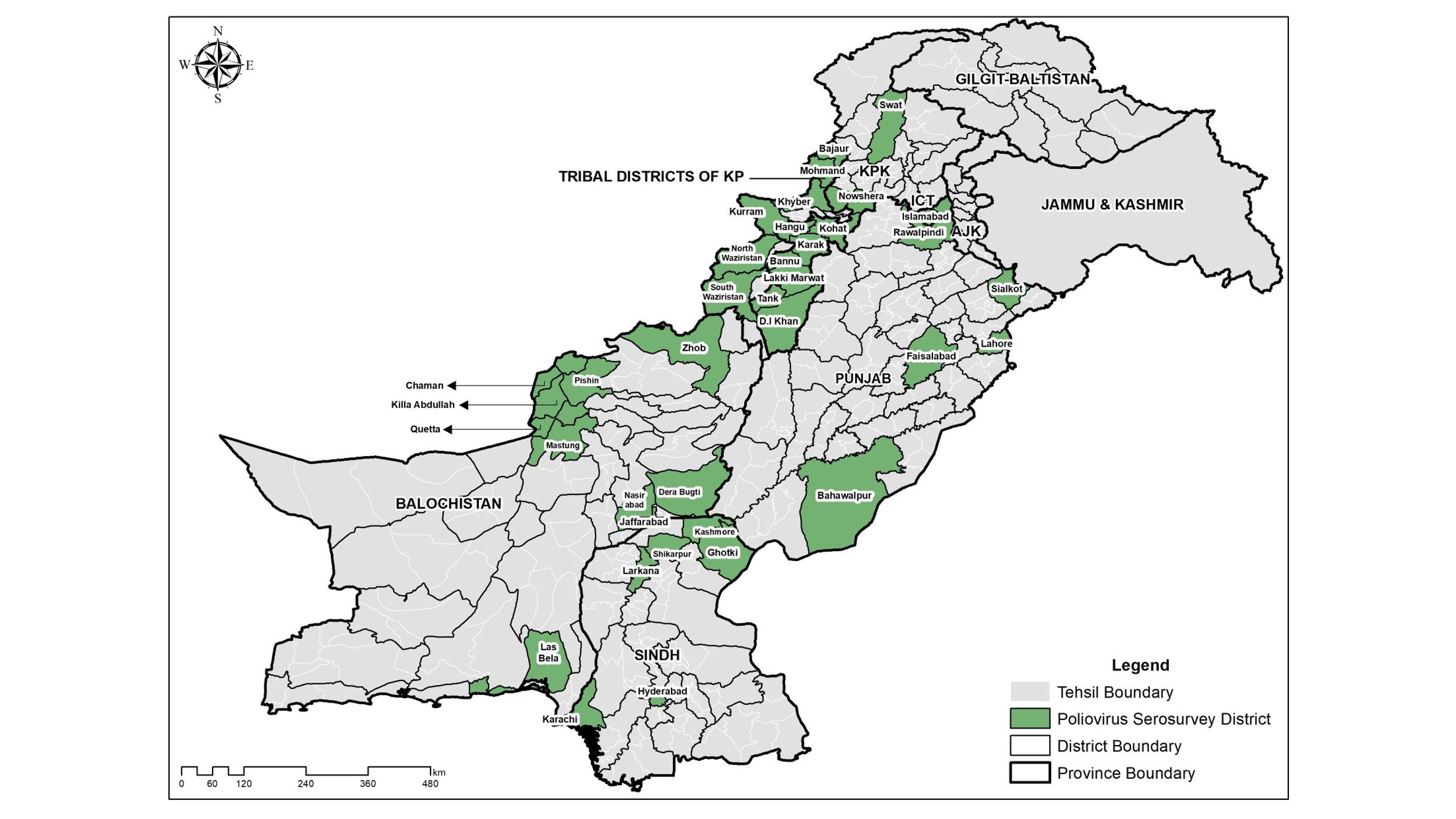
Figure S1. Geographic distribution of districts covered in the serosurvey

Table S1. District-wise demographic indicators, vaccination history of the study population

|  |  | **N** | **Age** | | **Gender of Child** | | **Child ever received IPV** | | **Total OPV doses (RI + SIAs)** | | **Immunization status** | | |
| --- | --- | --- | --- | --- | --- | --- | --- | --- | --- | --- | --- | --- | --- |
|  |  |  | **6-11 months** | **12-23 months** | **Male** | **Female** | **Yes** | **No** | **<4** | **>=4** | **Not immunized** | **Partially immunized** | **Fully immunized** |
| **Province** | **District** |  |  |  |  |  |  |  |  |  |  |  |  |
| Punjab | Rawalpindi | 457 | 233 (51.0%) | 224 (49.0%) | 225 (49.2%) | 232 (50.8%) | 435 (95.2%) | 22 ( 4.8%) | 248 (54.3%) | 209 (45.7%) | 3 ( 0.7%) | 414 (90.6%) | 40 ( 8.8%) |
|  | Lahore | 491 | 233 (47.5%) | 258 (52.5%) | 237 (48.3%) | 254 (51.7%) | 294 (59.9%) | 197 (40.1%) | 389 (79.2%) | 102 (20.8%) | 15 ( 3.1%) | 199 (40.5%) | 277 (56.4%) |
|  | Sialkot | 463 | 216 (46.7%) | 247 (53.3%) | 231 (49.9%) | 232 (50.1%) | 460 (99.4%) | 3 ( 0.6%) | 423 (91.4%) | 40 ( 8.6%) | 0 ( 0.0%) | 445 (96.1%) | 18 ( 3.9%) |
|  | Bahawalpur | 456 | 214 (46.9%) | 242 (53.1%) | 232 (50.9%) | 224 (49.1%) | 435 (95.4%) | 21 ( 4.6%) | 398 (87.3%) | 58 (12.7%) | 4 ( 0.9%) | 385 (84.4%) | 67 (14.7%) |
|  | Faisalabad | 440 | 210 (47.7%) | 230 (52.3%) | 215 (48.9%) | 225 (51.1%) | 392 (89.1%) | 48 (10.9%) | 355 (80.7%) | 85 (19.3%) | 4 ( 0.9%) | 328 (74.5%) | 108 (24.5%) |
| Sindh | Hyderabad | 483 | 214 (44.3%) | 269 (55.7%) | 251 (52.0%) | 232 (48.0%) | 362 (74.9%) | 121 (25.1%) | 275 (56.9%) | 208 (43.1%) | 39 ( 8.1%) | 308 (63.8%) | 136 (28.2%) |
|  | Ghotki | 462 | 231 (50.0%) | 231 (50.0%) | 248 (53.7%) | 214 (46.3%) | 200 (43.3%) | 262 (56.7%) | 403 (87.2%) | 59 (12.8%) | 178 (38.5%) | 147 (31.8%) | 137 (29.7%) |
|  | Shikarpur | 495 | 241 (48.7%) | 254 (51.3%) | 261 (52.7%) | 234 (47.3%) | 147 (29.7%) | 348 (70.3%) | 286 (57.8%) | 209 (42.2%) | 105 (21.2%) | 112 (22.6%) | 278 (56.2%) |
|  | Larkana | 489 | 251 (51.3%) | 238 (48.7%) | 281 (57.5%) | 208 (42.5%) | 343 (70.1%) | 146 (29.9%) | 414 (84.7%) | 75 (15.3%) | 9 ( 1.8%) | 272 (55.6%) | 208 (42.5%) |
|  | Kashmore | 473 | 229 (48.4%) | 244 (51.6%) | 245 (51.8%) | 228 (48.2%) | 128 (27.1%) | 345 (72.9%) | 369 (78.0%) | 104 (22.0%) | 80 (16.9%) | 105 (22.2%) | 288 (60.9%) |
|  | Karachi Central | 463 | 229 (49.5%) | 234 (50.5%) | 246 (53.1%) | 217 (46.9%) | 233 (50.3%) | 230 (49.7%) | 390 (84.2%) | 73 (15.8%) | 55 (11.9%) | 182 (39.3%) | 226 (48.8%) |
|  | Karachi East | 456 | 219 (48.0%) | 237 (52.0%) | 233 (51.1%) | 223 (48.9%) | 199 (43.6%) | 257 (56.4%) | 337 (73.9%) | 119 (26.1%) | 77 (16.9%) | 154 (33.8%) | 225 (49.3%) |
|  | Kemari | 468 | 233 (49.8%) | 235 (50.2%) | 259 (55.3%) | 209 (44.7%) | 221 (47.2%) | 247 (52.8%) | 348 (74.4%) | 120 (25.6%) | 88 (18.8%) | 164 (35.0%) | 216 (46.2%) |
|  | Korangi | 454 | 225 (49.6%) | 229 (50.4%) | 243 (53.5%) | 211 (46.5%) | 274 (60.4%) | 180 (39.6%) | 301 (66.3%) | 153 (33.7%) | 20 ( 4.4%) | 225 (49.6%) | 209 (46.0%) |
|  | Malir | 458 | 214 (46.7%) | 244 (53.3%) | 224 (48.9%) | 234 (51.1%) | 255 (55.7%) | 203 (44.3%) | 297 (64.8%) | 161 (35.2%) | 40 ( 8.7%) | 203 (44.3%) | 215 (46.9%) |
|  | Karachi South | 432 | 206 (47.7%) | 226 (52.3%) | 232 (53.7%) | 200 (46.3%) | 276 (63.9%) | 156 (36.1%) | 365 (84.5%) | 67 (15.5%) | 29 ( 6.7%) | 237 (54.9%) | 166 (38.4%) |
|  | Karachi West | 484 | 247 (51.0%) | 237 (49.0%) | 251 (51.9%) | 233 (48.1%) | 275 (56.8%) | 209 (43.2%) | 321 (66.3%) | 163 (33.7%) | 34 ( 7.0%) | 225 (46.5%) | 225 (46.5%) |
| Khyber Pakhtunkhwa | Peshawar | 494 | 237 (48.0%) | 257 (52.0%) | 276 (55.9%) | 218 (44.1%) | 381 (77.1%) | 113 (22.9%) | 348 (70.4%) | 146 (29.6%) | 33 ( 6.7%) | 303 (61.3%) | 158 (32.0%) |
|  | Khyber Agency | 475 | 238 (50.1%) | 237 (49.9%) | 239 (50.3%) | 236 (49.7%) | 297 (62.5%) | 178 (37.5%) | 283 (59.6%) | 192 (40.4%) | 23 ( 4.8%) | 219 (46.1%) | 233 (49.1%) |
|  | Bajaur Agency | 461 | 204 (44.3%) | 257 (55.7%) | 225 (48.8%) | 236 (51.2%) | 321 (69.6%) | 140 (30.4%) | 435 (94.4%) | 26 ( 5.6%) | 24 ( 5.2%) | 257 (55.7%) | 180 (39.0%) |
|  | Mohmand Agency | 461 | 219 (47.5%) | 242 (52.5%) | 228 (49.5%) | 233 (50.5%) | 334 (72.5%) | 127 (27.5%) | 365 (79.2%) | 96 (20.8%) | 50 (10.8%) | 258 (56.0%) | 153 (33.2%) |
|  | Kurram Agency | 481 | 222 (46.2%) | 259 (53.8%) | 248 (51.6%) | 233 (48.4%) | 359 (74.6%) | 122 (25.4%) | 407 (84.6%) | 74 (15.4%) | 1 ( 0.2%) | 286 (59.5%) | 194 (40.3%) |
|  | Hangu | 465 | 222 (47.7%) | 243 (52.3%) | 242 (52.0%) | 223 (48.0%) | 202 (43.4%) | 263 (56.6%) | 415 (89.2%) | 50 (10.8%) | 162 (34.8%) | 174 (37.4%) | 129 (27.7%) |
|  | Karak | 453 | 215 (47.5%) | 238 (52.5%) | 232 (51.2%) | 221 (48.8%) | 283 (62.5%) | 170 (37.5%) | 324 (71.5%) | 129 (28.5%) | 81 (17.9%) | 218 (48.1%) | 154 (34.0%) |
|  | Kohat | 452 | 219 (48.5%) | 233 (51.5%) | 227 (50.2%) | 225 (49.8%) | 304 (67.3%) | 148 (32.7%) | 374 (82.7%) | 78 (17.3%) | 50 (11.1%) | 250 (55.3%) | 152 (33.6%) |
|  | Swat | 474 | 209 (44.1%) | 265 (55.9%) | 251 (53.0%) | 223 (47.0%) | 413 (87.1%) | 61 (12.9%) | 381 (80.4%) | 93 (19.6%) | 6 ( 1.3%) | 361 (76.2%) | 107 (22.6%) |
|  | Nowshera | 479 | 223 (46.6%) | 256 (53.4%) | 246 (51.4%) | 233 (48.6%) | 331 (69.1%) | 148 (30.9%) | 409 (85.4%) | 70 (14.6%) | 35 ( 7.3%) | 279 (58.2%) | 165 (34.4%) |
|  | Dera Ismail Khan | 500 | 243 (48.6%) | 257 (51.4%) | 234 (46.8%) | 266 (53.2%) | 380 (76.0%) | 120 (24.0%) | 225 (45.0%) | 275 (55.0%) | 44 ( 8.8%) | 340 (68.0%) | 116 (23.2%) |
|  | Bannu | 495 | 245 (49.5%) | 250 (50.5%) | 256 (51.7%) | 239 (48.3%) | 116 (23.4%) | 379 (76.6%) | 361 (72.9%) | 134 (27.1%) | 237 (47.9%) | 84 (17.0%) | 174 (35.2%) |
|  | Lakki Marwat | 488 | 252 (51.6%) | 236 (48.4%) | 257 (52.7%) | 231 (47.3%) | 170 (34.8%) | 318 (65.2%) | 321 (65.8%) | 167 (34.2%) | 200 (41.0%) | 124 (25.4%) | 164 (33.6%) |
|  | Tank | 498 | 249 (50.0%) | 249 (50.0%) | 305 (61.2%) | 193 (38.8%) | 229 (46.0%) | 269 (54.0%) | 198 (39.8%) | 300 (60.2%) | 153 (30.7%) | 195 (39.2%) | 150 (30.1%) |
|  | N. Waziristan | 485 | 222 (45.8%) | 263 (54.2%) | 254 (52.4%) | 231 (47.6%) | 202 (41.6%) | 283 (58.4%) | 242 (49.9%) | 243 (50.1%) | 112 (23.1%) | 119 (24.5%) | 254 (52.4%) |
|  | S. Waziristan | 493 | 277 (56.2%) | 216 (43.8%) | 260 (52.7%) | 233 (47.3%) | 171 (34.7%) | 322 (65.3%) | 231 (46.9%) | 262 (53.1%) | 227 (46.0%) | 168 (34.1%) | 98 (19.9%) |
| Baluchistan | Quetta | 486 | 246 (50.6%) | 240 (49.4%) | 233 (47.9%) | 253 (52.1%) | 155 (31.9%) | 331 (68.1%) | 376 (77.4%) | 110 (22.6%) | 82 (16.9%) | 121 (24.9%) | 283 (58.2%) |
|  | Pishin | 464 | 239 (51.5%) | 225 (48.5%) | 222 (47.8%) | 242 (52.2%) | 120 (25.9%) | 344 (74.1%) | 305 (65.7%) | 159 (34.3%) | 193 (41.6%) | 82 (17.7%) | 189 (40.7%) |
|  | Killa Abdullah | 462 | 220 (47.6%) | 242 (52.4%) | 244 (52.8%) | 218 (47.2%) | 271 (58.7%) | 191 (41.3%) | 244 (52.8%) | 218 (47.2%) | 100 (21.6%) | 77 (16.7%) | 285 (61.7%) |
|  | Zhob | 465 | 240 (51.6%) | 225 (48.4%) | 251 (54.0%) | 214 (46.0%) | 106 (22.8%) | 359 (77.2%) | 395 (84.9%) | 70 (15.1%) | 247 (53.1%) | 61 (13.1%) | 157 (33.8%) |
|  | Dera Bugti | 437 | 207 (47.4%) | 230 (52.6%) | 249 (57.0%) | 188 (43.0%) | 4 ( 0.9%) | 433 (99.1%) | 383 (87.6%) | 54 (12.4%) | 303 (69.3%) | 2 ( 0.5%) | 132 (30.2%) |
|  | Nasirabad | 460 | 238 (51.7%) | 222 (48.3%) | 234 (50.9%) | 226 (49.1%) | 157 (34.1%) | 303 (65.9%) | 254 (55.2%) | 206 (44.8%) | 185 (40.2%) | 136 (29.6%) | 139 (30.2%) |
|  | Jaffarabad | 469 | 258 (55.0%) | 211 (45.0%) | 257 (54.8%) | 212 (45.2%) | 238 (50.7%) | 231 (49.3%) | 17 ( 3.6%) | 452 (96.4%) | 1 ( 0.2%) | 234 (49.9%) | 234 (49.9%) |
|  | Las Bela | 480 | 244 (50.8%) | 236 (49.2%) | 247 (51.5%) | 233 (48.5%) | 152 (31.7%) | 328 (68.3%) | 287 (59.8%) | 193 (40.2%) | 220 (45.8%) | 149 (31.0%) | 111 (23.1%) |
|  | Mastung | 450 | 230 (51.1%) | 220 (48.9%) | 224 (49.8%) | 226 (50.2%) | 221 (49.1%) | 229 (50.9%) | 159 (35.3%) | 291 (64.7%) | 45 (10.0%) | 202 (44.9%) | 203 (45.1%) |
|  | Chaman | 459 | 220 (47.9%) | 239 (52.1%) | 239 (52.1%) | 220 (47.9%) | 154 (33.6%) | 305 (66.4%) | 284 (61.9%) | 175 (38.1%) | 124 (27.0%) | 91 (19.8%) | 244 (53.2%) |
| Islamabad Capital Territory | Islamabad | 470 | 229 (48.7%) | 241 (51.3%) | 257 (54.7%) | 213 (45.3%) | 409 (87.0%) | 61 (13.0%) | 259 (55.1%) | 211 (44.9%) | 15 ( 3.2%) | 365 (77.7%) | 90 (19.1%) |

**Table S2. District-wise seroprevalence of poliovirus types 1, 2, and 3 among children aged 6–11 months and 12–23 months**

|  |  | **Poliovirus type 1** | | **Poliovirus type 2** | | **Poliovirus type 3** | |
| --- | --- | --- | --- | --- | --- | --- | --- |
| **Province** | **District** | **6-11 months** | **12-23 months** | **6-11 months** | **12-23 months** | **6-11 months** | **12-23 months** |
| Punjab | Rawalpindi | 98.7 (97.2,100.2) | 98.2 (96.4,100) | 55.7 (49.3,62.1) | 69.1 (63,75.2) | 98.3 (96.6,100) | 98.6 (97.1,100.2) |
|  | Lahore | 98.7 (97.2,100.2) | 98.4 (96.9,99.9) | 32.8 (26.7,38.9) | 46.1 (40,52.2) | 95.2 (92.4,98) | 96.5 (94.2,98.7) |
|  | Sialkot | 97.7 (95.7,99.7) | 99.6 (98.8,100.4) | 60.1 (53.5,66.7) | 69 (63.2,74.8) | 94.4 (91.3,97.5) | 99.2 (98.1,100.3) |
|  | Bahawalpur | 97.1 (94.8,99.4) | 99.2 (98,100.4) | 32.5 (26.1,38.9) | 46.2 (39.8,52.6) | 89 (84.7,93.2) | 97 (94.9,99.2) |
|  | Faisalabad | 98 (96,100) | 99.5 (98.6,100.4) | 41.2 (34.3,48.1) | 55 (48.4,61.6) | 96.5 (93.9,99) | 95 (92.1,97.9) |
| Sindh | Hyderabad | 97.5 (95.3,99.7) | 99.6 (98.9,100.3) | 25.4 (19.4,31.4) | 47.9 (41.9,53.9) | 93 (89.5,96.6) | 97 (94.9,99) |
|  | Ghotki | 97.8 (95.9,99.7) | 97 (94.8,99.2) | 23.3 (17.8,28.8) | 30.9 (24.9,36.9) | 84.6 (79.9,89.3) | 91.3 (87.7,95) |
|  | Shikarpur | 98.3 (96.7,99.9) | 96.1 (93.7,98.5) | 51 (44.7,57.3) | 54.7 (48.6,60.8) | 85.1 (80.6,89.6) | 90.2 (86.5,93.8) |
|  | Larkana | 99.6 (98.8,100.4) | 99.1 (97.9,100.3) | 50.2 (43.7,56.7) | 51.6 (45.1,58.1) | 94.4 (91.4,97.4) | 93.8 (90.6,96.9) |
|  | Kashmore | 90.3 (86.4,94.2) | 94.2 (91.3,97.1) | 26.5 (20.7,32.3) | 32.6 (26.7,38.5) | 82.7 (77.8,87.7) | 79.8 (74.7,84.8) |
|  | Karachi Central | 96.5 (94.1,98.9) | 100 (100,100) | 24 (18.5,29.5) | 37.5 (31.3,43.7) | 93.4 (90.2,96.7) | 95.7 (93.1,98.3) |
|  | Karachi East | 97.7 (95.7,99.7) | 97.9 (96.1,99.7) | 47.2 (40.6,53.8) | 54.7 (48.3,61.1) | 91.3 (87.5,95) | 92.4 (89,95.8) |
|  | Kemari | 97.4 (95.3,99.5) | 97 (94.8,99.2) | 45 (38.6,51.4) | 60.5 (54.2,66.8) | 91.8 (88.2,95.3) | 92.7 (89.4,96.1) |
|  | Korangi | 92.6 (89.1,96.1) | 98.1 (96.3,99.9) | 39.1 (32.6,45.6) | 63.3 (56.8,69.8) | 90.2 (86.3,94.2) | 90.2 (86.3,94.2) |
|  | Malir | 97.1 (94.8,99.4) | 98.7 (97.3,100.1) | 35.7 (29.2,42.2) | 50.6 (44.2,57) | 88.9 (84.6,93.2) | 92.8 (89.5,96.1) |
|  | Karachi South | 97.1 (94.8,99.4) | 100 (100,100) | 32.5 (26.1,38.9) | 52.7 (46.2,59.2) | 94.7 (91.6,97.7) | 96.9 (94.6,99.2) |
|  | Karachi West | 96.3 (93.9,98.7) | 98.7 (97.3,100.1) | 45.9 (39.7,52.1) | 63.7 (57.6,69.8) | 95.1 (92.4,97.8) | 99.2 (98,100.3) |
| Khyber Pakhtunkhwa | Peshawar | 97.7 (95.7,99.7) | 99.6 (98.8,100.4) | 54.6 (48,61.2) | 83.6 (78.9,88.3) | 94 (90.9,97.2) | 93.9 (90.8,96.9) |
|  | Khyber Agency | 97.3 (95.2,99.4) | 99.6 (98.7,100.5) | 71.7 (65.8,77.6) | 85.6 (81,90.2) | 90.7 (86.9,94.5) | 94.8 (91.9,97.7) |
|  | Bajaur Agency | 94.6 (91.5,97.7) | 96.4 (94.1,98.7) | 41.1 (34.3,47.9) | 49.2 (43,55.4) | 88.1 (83.6,92.6) | 94.4 (91.5,97.3) |
|  | Mohmand Agency | 93.3 (89.9,96.7) | 97.5 (95.5,99.5) | 45 (38.2,51.8) | 62.4 (56.2,68.6) | 89 (84.7,93.2) | 91.1 (87.5,94.8) |
|  | Kurram Agency | 94.3 (91.2,97.4) | 96.9 (94.8,99) | 33 (26.7,39.3) | 52.7 (46.6,58.8) | 93.9 (90.6,97.1) | 95.3 (92.7,97.9) |
|  | Hangu | 85.8 (81.1,90.5) | 90.5 (86.7,94.3) | 27.4 (21.4,33.4) | 30.6 (24.7,36.5) | 75.5 (69.7,81.3) | 84.5 (79.8,89.2) |
|  | Karak | 82.8 (77.6,88) | 89.8 (85.9,93.7) | 11.3 (6.9,15.7) | 13.6 (9.2,18) | 73 (66.9,79.1) | 82.2 (77.3,87.1) |
|  | Kohat | 94.6 (91.5,97.7) | 94.7 (91.8,97.6) | 33.2 (26.7,39.7) | 46.5 (40,53) | 88.8 (84.4,93.1) | 91.7 (88.1,95.3) |
|  | Swat | 98 (96.1,99.9) | 100 (100,100) | 40.6 (33.8,47.4) | 48.3 (42.2,54.4) | 98.5 (96.8,100.2) | 99.2 (98.2,100.3) |
|  | Nowshera | 98.5 (96.8,100.2) | 99.6 (98.8,100.4) | 35.5 (28.9,42.1) | 58.1 (51.9,64.3) | 94.6 (91.5,97.7) | 98 (96.2,99.7) |
|  | Dera Ismail Khan | 99.2 (98.1,100.3) | 99.6 (98.8,100.4) | 86.4 (82.1,90.7) | 87.5 (83.5,91.5) | 95 (92.3,97.8) | 96.1 (93.7,98.5) |
|  | Bannu | 95.9 (93.4,98.4) | 96 (93.5,98.5) | 70.8 (65.1,76.5) | 77.3 (72.1,82.5) | 86.8 (82.6,91.1) | 92.7 (89.5,96) |
|  | Lakki Marwat | 95.4 (92.7,98.1) | 96.9 (94.7,99.1) | 69.6 (63.7,75.5) | 72.8 (67,78.6) | 89.5 (85.5,93.4) | 92.1 (88.6,95.6) |
|  | Tank | 97.2 (95.1,99.3) | 100 (100,100) | 76.7 (71.4,82) | 79.9 (74.9,84.9) | 92.8 (89.5,96) | 91.6 (88.1,95) |
|  | N. Waziristan | 97.3 (95.1,99.5) | 98.5 (97,100) | 56.8 (50.2,63.4) | 64.3 (58.5,70.1) | 90.5 (86.6,94.3) | 95.1 (92.4,97.7) |
|  | S. Waziristan | 93.3 (90.3,96.3) | 91.2 (87.3,95.1) | 70.5 (65,76) | 69.3 (63,75.6) | 85.8 (81.6,90) | 90.7 (86.8,94.7) |
| Baluchistan | Quetta | 96.6 (94.3,98.9) | 96.1 (93.6,98.6) | 64.6 (58.5,70.7) | 58.8 (52.5,65.1) | 90.3 (86.5,94.1) | 89.3 (85.3,93.3) |
|  | Pishin | 92.6 (89.2,96) | 90.8 (86.9,94.7) | 41.1 (34.7,47.5) | 41.5 (34.9,48.1) | 83.5 (78.8,88.3) | 85.7 (81,90.4) |
|  | Killa Abdullah | 75.1 (69.3,80.9) | 77.1 (71.7,82.5) | 36.6 (30.1,43.1) | 36.9 (30.7,43.1) | 68.5 (62.3,74.8) | 66.5 (60.5,72.6) |
|  | Zhob | 82 (77,87) | 87.5 (83.1,91.9) | 19.3 (14.2,24.4) | 17.1 (12.1,22.1) | 68.9 (62.8,74.9) | 79.2 (73.7,84.6) |
|  | Dera Bugti | 88.2 (83.7,92.7) | 85.9 (81.4,90.4) | 26.6 (20.5,32.7) | 29.1 (23.2,35) | 74.4 (68.4,80.4) | 76.2 (70.7,81.8) |
|  | Nasirabad | 91.6 (88.1,95.1) | 93.7 (90.5,96.9) | 25.2 (19.7,30.7) | 34.4 (28.1,40.7) | 80.3 (75.2,85.3) | 84.2 (79.3,89) |
|  | Jaffarabad | 95.8 (93.2,98.4) | 95.7 (92.9,98.5) | 21.2 (16,26.4) | 27.1 (21,33.2) | 86.9 (82.5,91.2) | 87.9 (83.5,92.4) |
|  | Las Bela | 93 (89.8,96.2) | 93.6 (90.5,96.7) | 41.2 (35,47.4) | 53.8 (47.4,60.2) | 77.8 (72.5,83) | 89 (85,93) |
|  | Mastung | 93.4 (90.2,96.6) | 92.3 (88.8,95.8) | 63.8 (57.6,70) | 59.5 (53,66) | 89.5 (85.5,93.5) | 87.3 (82.9,91.7) |
|  | Chaman | 83.5 (78.4,88.6) | 87.4 (83,91.8) | 50 (43.2,56.8) | 56.5 (50,63) | 72.3 (66.2,78.5) | 82.1 (77,87.1) |
| Islamabad Capital Territory | Islamabad | 98.6 (97,100.2) | 99.1 (97.9,100.3) | 56.1 (49.4,62.8) | 73.2 (67.4,79) | 95.8 (93,98.5) | 97.3 (95.2,99.4) |
